# Urinary Tract Infection Caused by Capnophilic *Escherichia coli*

**DOI:** 10.3201/eid1407.071053

**Published:** 2008-07

**Authors:** Daniel Tena, Alejandro González-Praetorius, Juan Antonio Sáez-Nieto, Sylvia Valdezate, Julia Bisquert

**Affiliations:** * University Hospital of Guadalajara, Guadalajara, Spain; † Instituto de Salud Carlos III, Majadahonda, Madrid, Spain

**Keywords:** Escherichia coli, capnophilic, carbon dioxide-dependent strain, urinary tract infection, sterile pyuria, letter

**To the Editor:** Increased atmospheric CO_2_ concentrations promote the growth of fastidious microorganisms. However, the possibility that a strain of *Escherichia coli* can be CO_2_ dependent is exceptional (*1*).

An isolate of capnophilic *E. coli* was responsible for a urinary tract infection (UTI) in a 77-year-old woman at the University Hospital of Guadalajara (Spain) in November 2002. Urine was cultured on a cystine-lactose-electrolyte-deficient agar plate and incubated at 37°C in an atmosphere containing 6% CO_2_ for 1 day. After 24 hours, the culture yielded gram-negative rods (>10^5^ CFU/mL) in pure culture. The organism was motile, catalase positive, and oxidase negative. The strain could not be identified by using the MicroScan WalkAway-40 system (DadeBerhing, Inc., West Sacramento, CA, USA). A subculture was performed, and the organism did not grow on sheep blood agar and MacConkey agar plates at 37°C in ambient air. However, a subculture incubated at 37°C for 24 hours in an atmosphere of 6% CO_2_ produced smooth colonies 2–3 mm in diameter on sheep blood agar and MacConkey agar plates. The organism fermented lactose, and the indole reaction (BBL DrySlidet, Becton Dickinson Co., Sparks, MD, USA) performed on sheep blood agar was negative. The strain grew well on Schaedler agar plates after anaerobic incubation for 48 hours. The isolate remained capnophilic after 5 subcultures. The strain was identified as *E. coli* by using the Biolog GN2 panel (Biolog, Inc., Hayward, CA, USA) (100%, T = 0,534), after incubation of the panel in an atmosphere containing 6% CO_2_ for 1 day. The API 20E system (bioMérieux, Marcy-l’Etoile, France) according to the manufacturer’s instructions without C0_2_ incubation also identified *E. coli* (profile 5004512). The identification was confirmed by means of 16S rDNA sequence analysis (1,472 bp obtained by PCR amplification by a previously reported method [*2*]), which showed 99% similarity with *E. coli* sequence (GenBank accession no. CP000802). The 16S sequence showed similarity with *Shigella* species; however, this identification was not considered because the strain fermented lactose on MacConkey agar and agglutinations with *Shigella* antiserum were negative. The original 16S rDNA sequence was deposited in GenBank (accession no. EU555536).

The antimicrobial drug susceptibility profile was determined by incubating Mueller-Hinton agar plates at 37°C in an atmosphere containing 6% CO_2_ by the disk diffusion method, according to National Committee for Clinical Laboratory Standards recommendations (*3*). The isolate was susceptible to ampicillin, amoxicillin/clavulanic acid, piperacillin, cefazolin, cefuroxime, cefotaxime, nitrofurantoin, fosfomycin, trimethoprim-sulfamethoxazole, gentamicin, tobramycin, amikacin, norfloxacin, and ciprofloxacin. MICs were obtained for the following antimicrobial agents with the E-test method (AB Biodisk, Solna, Sweden), performed on Mueller-Hinton agar plates incubated in a 6% CO_2_ atmosphere: ampicillin (1.5 μg/mL), amoxicillin (3 μg/mL), cefotaxime (0.064 μg/mL), imipenem (0.094 μg/mL), piperacillin (2 μg/mL), and ciprofloxacin (0.008 μg/mL).

*E. coli* is the most common pathogen among patients with uncomplicated UTIs (*4*). Two cases of UTIs due to carbon dioxide–dependent strains of *E. coli* have been reported (*1*). The mechanisms for development of CO_2_ dependence are unknown (*5*). CO_2_ can play a role in the growth of *E. coli* as a substrate for carboxylation reactions (*6*). Other members of the family *Enterobacteriaceae* (such as some strains of *Klebsiella* spp.) and other organisms (such as *Staphylococcus aureus*)*,* can have similar requirements (*7*,*8*).

There is not 1 best way of performing urine cultures. Guidelines for the diagnosis of UTI includes the use of sheep blood agar and either MacConkey agar or a similar selective medium for routine urine culture. The plates should be incubated overnight (at least 16 hours) at 37°C in ambient air; alternatively, the blood agar plate can be incubated in elevated (3%–8%) CO_2_ (*9*). For fastidious microorganisms, chocolate agar can be added to the MacConkey agar and the plates incubated in 5% CO_2_ for 2 days (*9*).

The real incidence of these infections is unknown, but the rarity of these strains suggests that the incidence is low. However, the real incidence of UTI caused by capnophilic *E. coli* may be underestimated because urine cultures are not usually incubated in CO_2._ In addition, urine cultures are not performed for many women with uncomplicated cystitis. Other fastidious uropathogens such as *Haemophilus influenzae* and *H. parainfluenzae,* also require special media and incubation in an atmosphere of CO_2_ (*9*). The low frequency of these strains suggests that incubation of routine urine cultures in an atmosphere containing CO_2_ is not necessary. Incubation in CO_2_ should be ordered only if the patient has pyuria and a previous negative urine culture after incubation in ambient air or if the patient is unresponsive to empiric therapy and routine urine culture is negative. Good clinician–laboratory communication is vital. Further studies should be performed to ascertain the real incidence of UTIs caused by capnophilic strains of *E. coli.*

Because no breakpoints are available for antimicrobial agents against capnophilic strains of *E. coli*, we used published interpretative criteria or *Enterobacteriaceae* (*3*). The strain was susceptible to all antimicrobial agents that we tested. The impact of CO_2_ on the susceptibility of capnophilic strains of *E. coli* is unknown. Susceptibility of some antimicrobial agents such as quinolones can be influenced by the pH change and enhanced growth that occur during CO_2_ incubation when testing capnophilic organisms (*10*).
